# Pulsed-control plasma-activated water: An emerging technology to assist ultrasound for fresh-cut produce washing

**DOI:** 10.1016/j.ultsonch.2023.106739

**Published:** 2023-12-21

**Authors:** Jiayi Wang, Yincang Cui, Minwei Zhang, Liang Wang, Aihemaitijiang Aihaiti, Ruxianguli Maimaitiyiming

**Affiliations:** a Xinjiang Key Laboratory of Biological Resources and Genetic Engineering, College of Life Science & Technology, Xinjiang University, Urumqi 830046, China; b Physics and Chemistry Analysis Center, Xinjiang University, Urumqi 830046, China; c College of Food and Chemical Engineering, Shaoyang University, Shaoyang 422000, China

**Keywords:** Hurdle, Ultrasound, Minimal processing, Plasma-activated water

## Abstract

In this study, we explored the use of plasma-activated water (PAW) in combination with ultrasound (US) for food disinfection. Our research introduces a novel approach that utilizes a pulsed-control (PC) method to modify the PAW. The resulting PCPAW exhibits significantly higher concentrations of key reactive oxygen and nitrogen species (RONS) compared to conventional PAW. The disinfection efficacy of US–PCPAW against fresh-cut lettuce was compared to that of US-PAW, US, and PCPAW. The combination of US and PCPAW was highly effective in reducing food-borne pathogens, surpassing single treatments in count reduction and minimizing cross-contamination. Furthermore, our study demonstrates that US–PCPAW effectively controls browning appearance without compromising sensory attributes. These findings suggest that PCPAW, as a novel disinfectant, can be a valuable addition to US to enhance the quality and safety of fresh-cut produce.

## Highlights

• US-PCPAW showed the highest pathogen reduction (1.1–1.9 log CFU/g).
• The lowest pathogen cross-contamination incidence (27–33%) was achieved by US-PCPAW.
• US-PCPAW was effective to inactivate PPO and POD to control browning in fresh-cut lettuce.

## 1 Introduction

Fresh fruits and vegetables are rich in vitamins, minerals, and dietary fiber. Given the convenience and variety of fresh-cut produce, the fresh-cut industry has emerged as a significant contributor to the income generated in the agricultural sector [1]. However, because fresh-cut produce is typically consumed raw, there is a risk of foodborne disease outbreaks, particularly due to foodborne pathogen contamination [2]. Among various food-borne infections, *Salmonella* occurs the most frequently, followed by *Escherichia coli* O157:H7 [3], [4]. Additionally, when fresh-cut produce is cut, phenols are released from cells. These phenols react with oxygen, facilitated by polyphenol oxidase (PPO) and peroxidase (POD), leading to the formation of quinone compounds. This process results in a browning appearance that adversely affects sensory color [5]. Therefore, disinfection of foodborne pathogens and control of browning of fresh-cut produce using strategies that do not destroy nutritional compounds, such as non-thermal methods, are essential to the fresh-cut industry [5].

Low-frequency ultrasound (US) has been applied as a non-thermal food processing method to disinfect fresh-cut produce and control browning [5]. However, the efficacy of US alone is limited, and it has been suggested to be improved in combination with other methods [6]. US is generally combined with chemical compounds, such as chlorine-based sanitizers [6], acidic electrolyzed water [7], and peracetic acid [8]. Recently, the rinsing stage (removing the residue after washing with a sanitizer) was modified to improve the efficacy of US-chemical washing. Aqueous ozone has been utilized as an alternative to tap water to significantly enhance the efficacy of US-free chlorine against *Escherichia coli* O157:H7 and *Salmonella* Typhimurium in fresh-cut lettuce [5]. Wang et al. [8] combined US-peracetic acid with aerosolized ascorbic acid to process cherry tomatoes and found that this combination led to an additional 0.7–0.9 and 0.6–0.8 log CFU/g of *E. coli* O157:H7 and *S*. Typhimurium, respectively, in comparison with US-peracetic acid. PPO and POD are responsible for fresh-cut produce browning, and the inactivation activity of US-ascorbic acid [9], US-modified atmosphere packaging [10], and US-natural products [11] against these two enzymes was significantly higher than that of a single treatment. Evidently, a novel method that can be combined with US to process fresh-cut produce is required.

Plasma-activated water (PAW) is an innovative nonthermal disinfection method with a wide range of applications, particularly in enhancing the safety and viability of fresh-cut produce. Recent research has illuminated PAW’s potential by demonstrating its ability to effectively inhibit PPO activity and significantly reduce aerobic mesophilic counts (AMC) [12]. Further studies, such as those focused on kale and spinach, have shown that PAW can significantly reduce the presence of *E. coli*; however, the chlorophyll content is reduced [13].

Atmospheric-pressure plasma is commonly employed for preparing PAW because of its cost-effectiveness, utilizing readily available air as the feeding gas. During plasma discharge, the gaseous-air phase interacts with the liquid phase, leading to ionization and the creation of short-lived reactive oxygen–nitrogen species (RONS) within the water. The resulting stable RONS include ozone, H_2_O_2_, nitrate, and nitrite [14]. Notably, the disinfection effectiveness of PAW is directly correlated with the concentrations of these four compounds [15].

To optimize PAW, researchers have investigated factors such as discharge voltage and preparation time at a constant plasma discharge frequency [15]. For instance, Esua et al. [16] identified an optimal discharge voltage of 66 V for the preparation of PAW. This optimization significantly improved its disinfection efficacy, especially against *S.* Typhimurium in grass carp, when combined with US. Recently, a novel approach to PAW preparation, known as pulsed-control PAW (PCPAW), was introduced. The PCPAW involves adjusting the plasma discharge frequency to enhance the concentration of RONS [17]. Aihemaitijiang et al. [17] observed that a discharge frequency of 200 Hz resulted in the highest concentrations of ozone, H_2_O_2_, nitrate, and nitrite (5.0-, 3.6-, 8.1-, and 1.5-fold, respectively) when compared to PAW prepared under the original plasma frequency of 10 kHz.

Despite these advancements, a critical gap in knowledge exists concerning the effectiveness of combining US with PCPAW. Therefore, the objective of this study was to combine US with PCPAW (US-PCPAW) and compare it with conventional US-PAW to disinfect inoculated foodborne pathogens, naturally occurring microbes, and the browning-related properties of fresh-cut produce. Iceberg lettuce, a model generally used in fresh-cut studies, was selected for analysis.

## 2 Materials and methods

### 2.1 Sample preparation

Iceberg lettuce was purchased from a local market on the day of the experiment, and the outer and inner layers were removed for further rinsing with tap water (30 s) to remove any dirt. The leaves were cut into 3 × 3 cm pieces [18] and dewatered using a sterilized (75 % ethanol) manual salad spinner.

### 2.2 Inoculation

*E. coli* O157:H7 (NCTC12900), non-O157 *E. coli* (ATCC25922), and *S*. Typhimurium (ATCC14028) were selected for lettuce inoculation [4]. The two pathogens were cultured overnight in nutrient broth at 37 °C with shaking at 120 rpm. After washing with 0.85 % NaCl three times under centrifugation at 12000 *× g* [19], the cell precipitate was resuspended in 0.85 % NaCl and the concentration was adjusted to approximately 10^9^ CFU/mL. Lettuce samples were immersed in the bacterial suspension at a ratio of 1:20 (w/v) and stirred for 2 min [18]. The inoculated lettuce was transferred to a biological safety cabinet for air-drying. Then, the sample was stored at 4 °C for 12 h to ensure pathogen attachment, and an inoculated sample with 7.1 ± 0.2 log CFU/g, 6.9 ± 0.4 log CFU/g, and 7.2 ± 0.2 log CFU/g of *E. coli* O157:H7, non-O157 *E. coli*, and *S*. Typhimurium were obtained, respectively.

### 2.3 PAW preparation and characterization

A modified dielectric barrier discharge (DBD) plasma system (CTP-2000 KP, Suman, Nanjing, China) containing a plasma generator (with a fixed discharge frequency of 10 kHz), pulsed wave generator (used for frequency control), DBD chamber, voltage booster, and digital oscilloscope was used for PAW preparation. Detailed information on the plasma system, PAW preparation procedure, plasma output power calculation, RONS concentration, and physical properties (pH, conductivity, and ORP) of PCPAW and PAW was obtained from our previous study [17]. Because the increased temperature was associated with plasma discharge, the resulting PCPAW and PAW was immediately transferred to a tube and placed in liquid nitrogen to decrease the temperature to 4 ± 1 °C within 30 s. Although the highest RONS concentration was observed at 200 Hz [17], quality loss (browning spots) was observed in lettuce leaves after washing with 200 Hz PAW and was not observed when employing 50 Hz PAW. Thus, in this study, PAW and PCPAW were prepared at 10 kHz and 50 Hz, respectively.

### 2.4 Disinfection and microbiological analysis

The inoculated lettuce was transferred into a sterilized beaker, which was placed in an ultrasound-assisted (28 kHz, 300 W) [5] water bath. The PAW and PCPAW were immediately poured into a beaker at a ratio of 1:10 (w/v). During disinfection, a stirrer (JB-80SH; XiuLab, Beijing, China) was placed in the beaker and stirred at 120 rpm to simulate water flow on the processing line [18]. The washing solution for the US treatment alone was distilled water. After disinfection for 1 min, the sample was rinsed with tap water, as described by Sun et al. [5], to remove PAW residue. The samples were then dewatered using a sterilized manual salad spinner. The sample was then transferred to a sterilized stomacher bag containing 0.85 % NaCl at a ratio of 9:1 (v/w) and homogenized for 2 min. A serially diluted bacterial suspension was cultured to quantify the counts of *E. coli* O157:H7, non-O157 *E. coli*, *S.* Typhimurium, aerobic mesophilic counts (AMC), aerobic psychrophilic counts (APC), and molds and yeasts (M&Y), as described by Wang et al. [6]. All results were expressed as log CFU/g. The disinfection efficacy of all treatments was expressed as count reduction based on the counts of the control.

### 2.5 Cross-contamination incidence

The incidence of cross-contamination was determined as described by Pablos et al. [20] with some modifications. The cell precipitate of the three pathogens in a sterilized tube was resuspended to the desired concentration (10^5^–10^6^ CFU/mL) using PAW, PCPAW, and distilled water within 10 s and then poured above the pathogen suspension into a beaker containing a lettuce sample that was not inoculated with the pathogen. The treatment procedure was performed as described in Section 2.4. Distilled water was used as control. The inoculated counts in the treatment group were divided by those in the control group and the obtained value was defined as the incidence of cross-contamination.

### 2.6 Pathogen cell membrane permeability

Cell membrane permeability was analyzed according to the method reported by Wang et al. [19]. The cell precipitate of the three pathogens was resuspended to 10^6^–10^7^ CFU/mL as described in Section 2.5, and disinfection was stopped by adding phosphate-buffered saline (pH 7.0; 1500 U/mL catalase (Yuanye, Shanghai, China), 15 mM L-histidine (Yuanye), 100 μM carboxy-PTIO (Yuanye), 5 mM Na_2_S_2_O_3_ (SCR, Shanghai, China)) at a ratio of 1:1 (v/v). After filtering through 0.22-µm filters, protein and alkaline phosphatase (AKP) contents were analyzed using test kits (Jiancheng, Nanjing, China), and the nucleotide content was measured at 260 nm.

### 2.7 Browning-related properties

#### 2.7.1 Browning potential and phenolic content analysis

The browning potential (BP) was analyzed as described by Vanden Abeele et al. [21] with minor modifications. A 10-g lettuce sample was immersed in liquid nitrogen for 30 s and subsequently transferred to an IKA analytical mill for processing for 30 s. The ground powder was dissolved in 20 mL 80 % methanol. After centrifugation for 10 min at 12000 *× g* at 4 °C, the absorbance of the supernatant was recorded at 320 nm, and the results were expressed as absorbance units per gram of sample (AU/g). The methanol-dissolved powder was placed in an ultrasound-assisted water bath for 30 min for complete extraction, followed by centrifugation for 10 min at 12000 *× g*. The phenolic content in the supernatant was quantified as described by Wang et al [4].

#### 2.7.2 PPO, POD, and phenylalanine ammonia-lyase (PAL)

Liquid nitrogen grounded powder (0.5 g) was dissolved in 2.5 mL of 0.1 M acetic acid-sodium acetate buffer (pH 5.5; 1 mM polyethylene glycol, 4 % crosslinking polyvingypyrrolidone, 1 % Triton X-100) and centrifuged at 12,000 × *g* at 4 °C for 20 min. The supernatant (0.5 mL) was mixed with 3 mL of 25 mM guaiacol solution and 200 μL of 0.5 M H_2_O_2_ solution, and absorbance was measured at 470 nm every minute. The increase in absorbance per minute of a 1-g sample was defined as one unit (U) of POD. For PPO analysis, 0.1 mL of supernatant was mixed with 4 mL of 50 mM acetic acid-sodium acetate buffer (pH 5.5) and 1 mL of 50 mM catechol solution, and the absorbance was measured at 420 nm every minute. The increase in absorbance per minute of a one-gram sample was defined as one unit (U) of PPO.

Grounded powder (0.5 g) was dissolved in 0.1 M of boric acid-borax buffer (pH 8.8; 4 % polyvinylpyrrolidone, 2 mM ethylenediaminetetraacetic acid, and 5 mM 2-mercaptoethanol) for PAL analysis. After centrifugation at 12,000 × *g* at 4 °C for 20 min, 1 mL of the supernatant was mixed with 6 mL acid-borax buffer and 1 mL of 20 mM *ι*-phenylalanine, and the absorbance was measured at 290 nm. An increase in absorbance per hour for a one-gram sample was defined as one unit (U).

### 2.8 Sensory quality

Sensory analyses of crispness, odor, and color (surface and cut-edge browning) were performed as previously described by Wang et al. [22] with minor modifications. Briefly, six panelists (three men and three women) were invited, and the score standards were as follows: 0, extremely unlikely; 5, acceptable threshold; and 10, extremely likely. The sample was placed on a white plate with a mark at the bottom and was reordered before evaluation. During the evaluation, only one person was allowed to enter the room, which was constructed according to ISO 8589:2007, and communication between the panelists was prohibited.

### 2.9 Statistical analysis

Differences between the means of the groups were evaluated using one-way analysis of variance and post-hoc Duncan’s multiple range test using SPSS v.20 (SPSS, Chicago, IL, USA). Statistical significance was set at *P* < 0.05. Each experiment was independently performed three times, and a sample treated with distilled water was used as the control.

## 3 Results and discussion

### 3.1 Cross-contamination prevention ability of different treatments

The physical and chemical properties of PAW and PCPAW are listed in Table 1. We observed increases in the output power, ORP, conductivity, and concentrations of ozone, H_2_O_2_, nitrate, and nitrite of 15 %, 10 %, 90 %, 144 %, 47 %, 217 %, and 41 %, respectively, while the frequency decreased from 10 kHz to 50 Hz, which is consistent with our previous report [17]. This was attributed to the increased output power, which led to a higher RONS concentration [17].

**Table 1:** Physical and chemical properties of PAW.

Parameter	Discharge frequency (Hz)	
	10 k	50
Output power	12.74 ± 1.46	14.70 ± 0.38
pH	3.36 ± 0.18	3.01 ± 0.12
ORP (mV)	455.33 ± 15.95	500.67 ± 17.50
Conductivity (μs/cm)	183.00 ± 8.00	347.00 ± 55.65
Ozone (mg/L)	1.08 ± 0.20	2.63 ± 0.44
Hydrogen peroxide (μM)	198.33 ± 25.17	290.83 ± 75.06
Nitrate (mg/L)	4.29 ± 0.20	13.60 ± 3.03
Nitrite (mg/L)	0.32 ± 0.06	0.45 ± 0.05

PAW, plasma-activated water; ORP, oxidation–reduction potential.

**Fig. 1:**
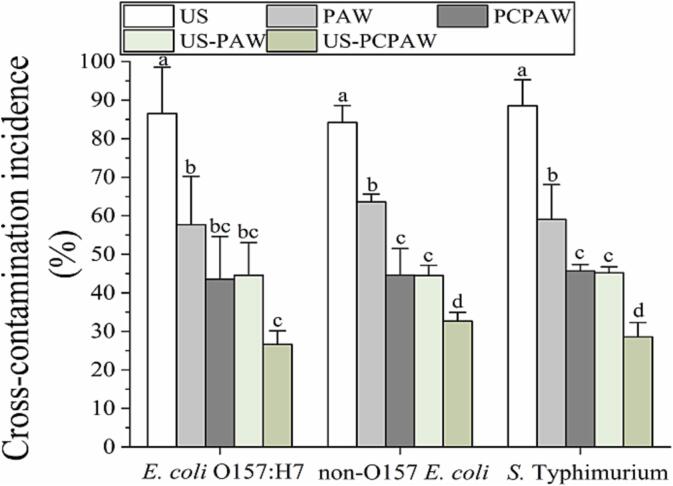
Cross-contamination prevention capacity of different treatments. Bars show mean ± standard deviation values, and different lowercase letters in the same group indicate significant differences (*P* < 0.05). US, ultrasound; PAW, plasma-activated water; PCPAW, pulsed-control plasma-activated water.

When fresh produce is submerged in washing water, pathogens present on the surface of the produce can leach into the circulating water, potentially contaminating other produce. This process can lead to cross-contamination and increase the risk of food safety incidents [23]. In this study, when US was used alone, the cross-contamination incidence for the three pathogens was 80–90 % (Fig. 1), which is consistent with the results of Wang et al. [6]. This result was attributed to the short processing time and limited efficacy of US. Costello et al. [24] showed that the inactivation efficacy of US against *E. coli* was less than 1 log CFU/mL even after treatment for 30 min. The representative gram-positive *Staphylococcus aureus* was only inactivated by 0.25 log CFU/mL after treatment with US for 30 min [25]. When employing PAW, the cross-contamination incidence was 58–64 %; however, the incidence further decreased to 44–46 % when using PCPAW, which is attributed to the increased RONS concentration in PCPAW compared to that in PAW (Table 1). When US was combined with PAW (US-PAW), the incidence of cross-contamination was consistent with that of PCPAW. The lowest incidence (27–33 %) was observed with US–PCPAW, which was significantly lower than that in the US.

### 3.2 Inactivation capacity of different treatments

**Fig. 2:**
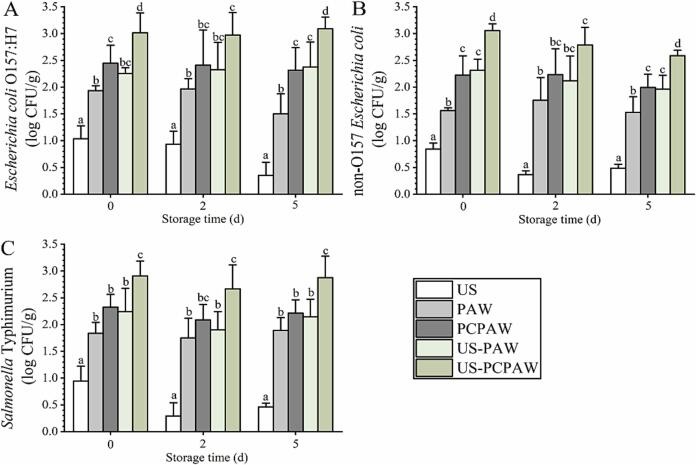
Inactivation capacity of different treatments against inoculated foodborne pathogens. (A) *Escherichia coli* O157:H7, (B) non-O157 *E. coli*, and (C) *S.* Typhimurium. Bars show mean ± standard deviation values, and different lowercase letters in the same group indicate significant differences (*P* < 0.05). US, ultrasound; PAW, plasma-activated water; PCPAW, pulsed-control plasma-activated water.

**Fig. 3:**
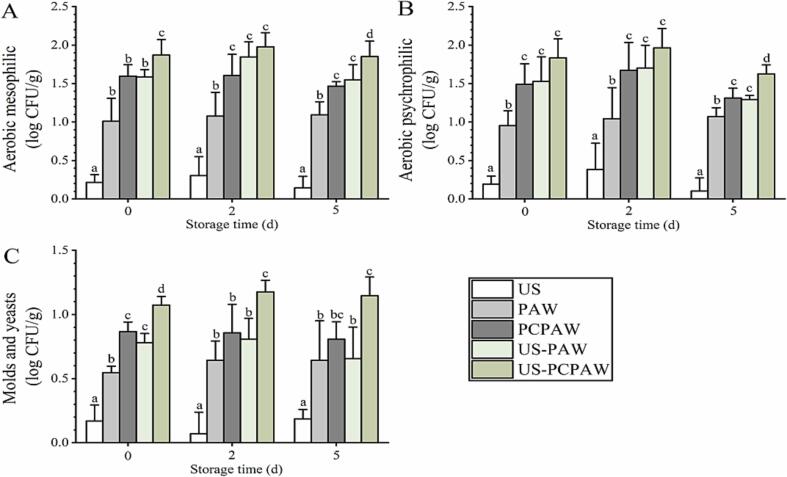
Inactivation capacity of different treatments against naturally present microbes. (A) aerobic mesophilic count, (B) aerobic psychrophilic count, and (C) number of molds and yeasts. Bars show mean ± standard deviation values, and different lowercase letters in the same group indicate significant differences (*P* < 0.05). US, ultrasound; PAW, plasma-activated water; PCPAW, pulsed-control plasma-activated water.

When using US alone, the three pathogens were inactivated by 0.8–1.0 log CFU/g (Fig. 2), consistent with previous reports [4], [5]. When using PAW and PCPAW, three pathogens were inactivated by 1.6–1.9 log CFU/g and 2.1–2.6 log CFU/g, respectively. After combining PAW with US, the efficacy was consistent with PCPAW, whereas the highest counts reduction was achieved by US–PCPAW, ranging from 2.9 to 3.1 log CFU/g. The highest count reduction was achieved by US-PCPAW during storage (2–5 d). When analyzing naturally present microbes, the count reduction achieved by US was less than 0.2 log CFU/g, significantly lower than that of PAW (0.6–1.0 log CFU/g) and PCPAW (0.9–1.6 log CFU/g) (Fig. 3). After combining PAW with US, its efficacy was found to be consistent with that of PCPAW. The highest count reduction was achieved by US-PCPAW, ranging from 1.1 to 1.9 log CFU/g. During storage (2–5 d), US-PCPAW still led the highest count reduction, inactivating the microbes at 1.2–2.0 log CFU/g and 1.2–1.9 log CFU/g on days 3 and 5, respectively.

**Fig. 4:**
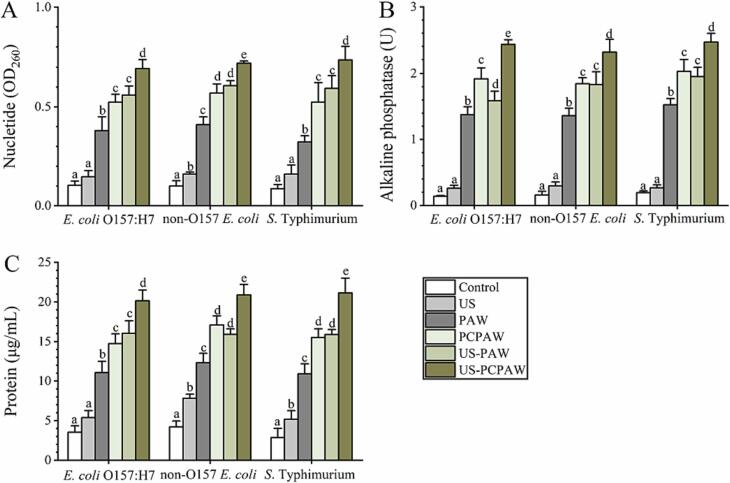
Inactivation capacity of different treatments against cell membrane properties in food-borne pathogen. (A) nucleotide, (B) alkaline phosphatase, and (C) protein content. Bars show mean ± standard deviation values, and different lowercase letters in the same group indicate significant differences (*P* < 0.05). US, ultrasound; PAW, plasma-activated water; PCPAW, pulsed-control plasma-activated water.

Evidently, US-PAW and US-PCPAW have a stronger disinfection efficacy than single treatments, based the analysis of cross-contamination incidence and pathogen inactivation capacity. US generates periodic cavitation liquid bubbles and local high temperatures (5500 K) and high pressures (50 MPa), and shear forces are formed as the bubbles rupture [26]. After 4 min of US treatment, water molecules were decomposed to H^+^ and OH^–^, which combined with H_2_O to form H_2_O_2_, simultaneously leading to oxidation and mechanical damage to the cell membrane [27]. However, the processing time in this study was only 1 min, indicating that US may cause physical damage to the cell membranes of these three pathogens. A previous review concluded that the antibacterial mechanism of action of PAW was to damage the cell membrane by breaking the bodies of peptidoglycans [14]. Therefore, the mechanism of action of US–PAW/PCPAW may be associated with cell membrane damage. Correspondingly, indicators related to cell membrane permeability (nucleotides, AKP, and proteins) were analyzed in this study. The results indicated that the efficacy of US–PAW against these three pathogens was significantly higher than that of US and PAW (Fig. 4). The highest leakage extent of AKP, protein, and nucleotides in the three pathogen membranes was observed with US-PCPAW, which was 1.2–1.3, 1.2–1.4, and 1.3–1.4-fold, respectively, compared to PCPAW.

These results indicate that the combination treatment can enhance inactivation efficacy by increasing the extent of membrane damage compared to a single treatment. PAW and PCPAW are acidic oxidizing disinfectants, and similar results were observed when US was combined with another acidic oxidizing solution. Akter et al. [7] combined slightly acidic electrolyzed water (SAEW) with US to process fresh-cut cauliflower, and an additional 0.8 and 1.0 log CFU/g reduction in AMC and M&Y, respectively, was observed when compared with SAEW alone. The disinfection efficacy of SAEW against *S.* Typhimurium in fresh-cut bell peppers was significantly improved in combination with US [28].

### 3.3 Effects of different treatments on sensory and browning-related properties of fresh-cut lettuce

**Fig. 5:**
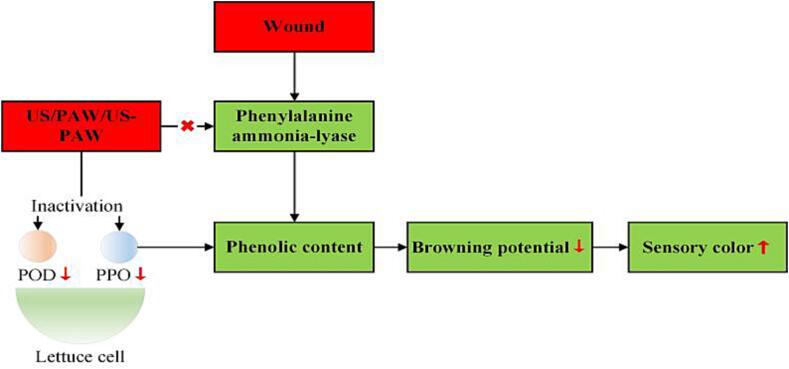
Browning process of fresh-cut produce. PPO, polyphenol oxidase; POD, peroxidase.

**Fig. 6:**
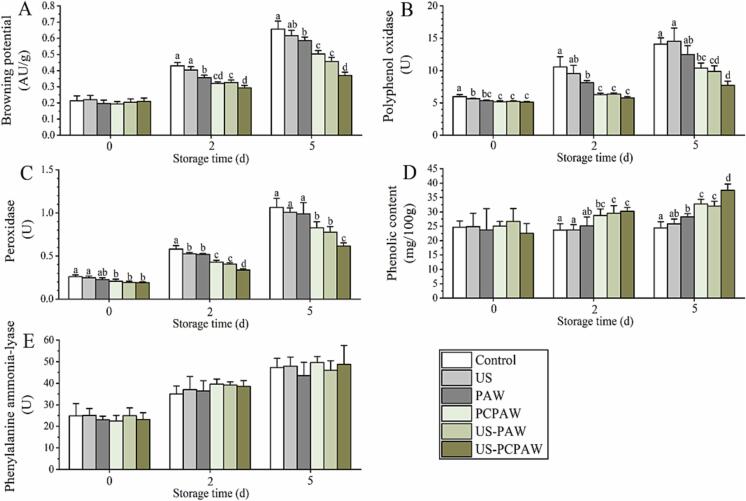
Effects of different treatments on browning-related parameters of fresh-cut lettuce. (A) browning potential, (B) polyphenol oxidase, (C) peroxidase, (D) phenolic, and (E) phenylalanine ammonia-lyase. Bars show mean ± standard deviation values, and different lowercase letters in the same group indicate significant differences (*P* < 0.05). US, ultrasound; PAW, plasma-activated water; PCPAW, pulsed-control plasma-activated water.

After cutting, the outflow of polyphenols from the wound reacts with PPO and POD under the influence of oxygen, forming quinones that negatively impact sensory color (Fig. 5). As ROS, O_3_ and H_2_O_2_ have been reported to inactivate PPO and POD to control browning in fresh-cut produce, and their efficacy is positively associated with ROS concentration [29], [30], [31]. Moreover, when the pH is below 4.0, PPO and POD activities can be inactivated, and their efficacy is negatively associated with pH [32]. In addition, US was found to inactivate PPO and POD and control browning in fresh-cut produce [33]. Using PAW as an acid sanitizer (pH < 4.0) containing ozone and H_2_O_2_, we hypothesized that the combination of US and PAW might improve the anti-browning effect compared to a single treatment. The results of the present study indicated that PPO and POD were significantly inactivated by PCPAW (Fig. 6B–C). During storage (2–5 d), the lowest PPO and POD activities were observed in the US–PCPAW group (Fig. 6B–C). Upon further analysis of the browning potential, the value of US-PCPAW was 67 % and 56 % of that of the control on days 2 and 5, respectively, which was significantly lower than that of US-PAW (Fig. 6A).

**Fig. 7:**
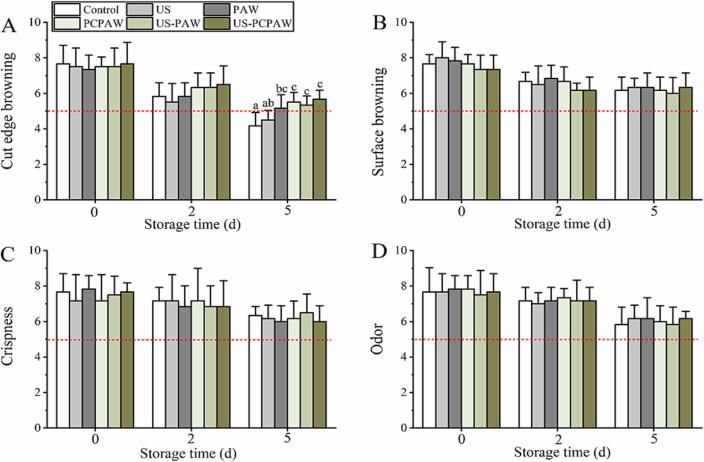
Effects of different treatments on sensory properties of fresh-cut lettuce. (A) cut edge browning, (B) surface browning, (C) crispness, and (D) odor. Bars show mean ± standard deviation values, and different lowercase letters in the same group indicate significant differences (*P* < 0.05). US, ultrasound; PAW, plasma-activated water; PCPAW, pulsed-control plasma-activated water.

Wounds can induce PAL expression, which is responsible for phenolic synthesis [34]. In this study, an increasing trend in PAL was observed on days 0–5, but no significant difference was observed between the groups (Fig. 6E). PAW and US, as a solution containing ROS and a physical stimulus to fresh produce, respectively, can induce a stress response that promotes phenolic synthesis; however, the processing time is at least 2 min [17], [35]. The processing time in this study was set to 1 min to meet the continuous processing line requirements [36], [37]. Thus, increased PAL activity during storage was induced by the wound instead of any other treatment (Fig. 5). When analyzing phenolic compounds, we found that the content in the US-PAW and US-PCPAW groups was significantly higher than that in the control and US groups (Fig. 6D), in contrast to the results for PPO and POD. This may be because the combination treatments showed higher PPO and POD inactivation capacities, which delayed the formation of quinone from the phenolic compounds [38]. When analyzing the sensory color, we found that the score of the cut edge in the control and US groups was below the acceptability threshold on day 5 (Fig. 7A), whereas the score observed in the other groups exceeded 5. In addition to the cut edge, the lettuce surface can also be damaged by ozone (>3.5 mg/L), leading to browning when the processing time exceeds 2 min [39]. As another oxidizing sanitizer, Salgado et al. [40] found that the combination of free chlorine (100 mg/L) and US for 1 min led to a lower sensory surface browning score in fresh-cut lettuce than in the control. In this study, sensory surface browning was not observed after processing (Fig. 7B), which may have been due to the short processing time (1 min) and low ozone concentration (1.1–2.6 mg/L). The other sensory properties (odor and crispness) were not negatively affected by any treatment on days 0–5.

## 4 Conclusion

In this study, we compared the efficacies of US, PAW, PCPAW, US-PAW, and US-PCPAW in terms of pathogen cross-contamination incidence, count reduction, sensory quality, and browning-related properties. PCPAW exhibited a higher concentration of RONS than PAW. Notably, when PCPAW and PAW were combined with US, the cross-contamination incidence decreased compared to the single treatments, with the lowest incidence (27–33 %) achieved by US-PCPAW. Moreover, the most substantial reduction in pathogen and naturally occurring microbial counts in lettuce occurred with the US-PCPAW treatment. The enhanced efficacy of US-PCPAW and US-PAW compared to single treatments can be attributed to greater damage to the cell membrane. Furthermore, PCPAW, US-PAW, and US-PCPAW significantly reduced PPO and POD activities. This reduction in enzyme activity led to decreased consumption of phenolic compounds, thereby inhibiting quinone formation, which, in turn, mitigated browning and improved sensory color quality. None of the treatments, either alone or in combination, had detrimental effects on sensory odor or crispness. Consequently, we propose US-PCPAW as an innovative hurdle technology for enhancing both the quality and microbial safety of fresh-cut produce.

## CRediT authorship contribution statement

**Jiayi Wang:** Conceptualization, Funding acquisition, Supervision, Writing – original draft, Writing – review & editing. **Yincang Cui:** Writing – review & editing. **Minwei Zhang:** Funding acquisition, Writing – review & editing. **Liang Wang:** Data curation, Funding acquisition. **Aihemaitijiang Aihaiti:** Data curation. **Ruxianguli Maimaitiyiming:** Writing – review & editing.

## Declaration of competing interest

The authors declare that they have no known competing financial interests or personal relationships that could have appeared to influence the work reported in this paper.
